# Robust Learning with Noisy Ship Trajectories by Adaptive Noise Rate Estimation

**DOI:** 10.3390/s23156723

**Published:** 2023-07-27

**Authors:** Haoyu Yang, Mao Wang, Zhihao Chen, Kaiming Xiao, Xuan Li, Hongbin Huang

**Affiliations:** Laboratory for Big Data and Decision, National University of Defense Technology, Changsha 410073, China; yanghaoyu20@nudt.edu.cn (H.Y.); wangmao@nudt.edu.cn (M.W.); chenzhihao22@nudt.edu.cn (Z.C.); kmxiao@nudt.edu.cn (K.X.); xuanli@nudt.edu.cn (X.L.)

**Keywords:** AIS data, deep learning, trajectory classification, label noise, robustness, noise rate adaptive learning, real-world data

## Abstract

Ship trajectory classification is of great significance for shipping analysis and marine security governance. However, in order to cover up their illegal fishing or espionage activities, some illicit ships will forge the ship type information in the Automatic Identification System (AIS), and this label noise will significantly impact the algorithm’s classification accuracy. Sample selection is a common and effective approach in the field of learning from noisy labels. However, most of the existing methods based on sample selection need to determine the noise rate of the data through prior means. To address these issues, we propose a noise rate adaptive learning mechanism that operates without prior conditions. This mechanism is integrated with the robust training paradigm JoCoR (joint training with co-regularization), giving rise to a noise rate adaptive learning robust training paradigm called A-JoCoR. Experimental results on real-world trajectories provided by the Danish Maritime Authority verified the effectiveness of A-JoCoR. It not only realizes the adaptive learning of the data noise rate during the training process, but also significantly improves the classification performance compared with the original method.

## 1. Introduction

With the rapid development of information technology, marine datasets are growing at an astonishing rate, driving the ocean into the era of big data. Marine data possesses characteristics such as large volume, diversity, and spatiotemporal properties, making it a typical application area for big data [1]. Among marine data, ship trajectory dataset is an important component, formed by collecting and recording a series of navigation information generated by ships during their voyages. These data form sequences with temporal and spatial attributes, composed in the order of collection time.

The Automatic Identification System (AIS) [2] is currently the most widely used global ship identification and tracking system in the field of maritime traffic. The AIS system encodes and broadcasts key information of ships (such as position, speed, heading, ship type, etc.) through transmitters and receivers on the ships. This information can be received and used by surrounding ships, shore-based stations, and satellites to monitor the real-time positions and navigation statuses of ships.

Through AIS, ships are able to perceive and recognize each other, taking timely evasive actions to reduce the risk of collisions at sea. AIS provides not only static information about the ships, such as length, width, and ship type, but also dynamic information including latitude, longitude, and acquisition time. These pieces of information can be used for predicting ship behavior [3,4,5,6] and trajectories [7,8], supporting maritime search and rescue systems [9,10], detecting fishing activities [11,12], as well as identifying anomalous behavior [13,14,15,16]. This greatly facilitates maritime operations and enhances maritime safety governance. Additionally, the ship type information in AIS plays a crucial role in shipping analysis, prevention of maritime terrorism, and combating maritime smuggling activities [17].

In order to better understand and utilize ship trajectory data, the task of ship trajectory classification has emerged. This task aims to construct classification models by extracting feature information embedded in trajectory data to accurately determine the types of different ship trajectories. This ship trajectory-based classification task has extensive application value and prospects. It can be used in maritime traffic management to assist in monitoring and controlling ship operations. In the field of maritime safety, it helps identify suspicious ship activities and prevent illegal behaviors such as terrorism. Moreover, it can provide decision-making support for marine resource management, promoting the sustainable development of fisheries and shipping industries.

However, it is important to note that AIS data presents certain challenges and issues in practical applications. Due to the susceptibility of AIS data to manipulation by vessel owners, some illicit vessels may intentionally falsify ship type information to conceal illegal fishing activities, espionage operations, or other unlawful behaviors. According to statistics, illegal fishing activities result in the capture of approximately 11 to 26 million metric tons of fish annually, accounting for 15% of global fish consumption [18]. Additionally, there are instances where certain countries may engage in malicious manipulation of AIS data, disguising reconnaissance vessels as neighboring fishing vessels, leading to security risks and geopolitical concerns.

Moreover, AIS data itself may contain recording or transmission errors, resulting in ship type information not matching the actual situation. Such data noise and errors pose challenges to ship trajectory-based classification tasks because incorrect ship type information increases the difficulty of detecting maritime illegal activities, posing a serious threat to maritime safety.

Trajectory classification methods based on deep learning typically assume that the ship types in the dataset are correctly labeled. However, this assumption is often difficult to meet in real-world scenarios. Deep learning models have powerful learning capabilities and can fit training sets with arbitrary label noise proportions [19]. However, the presence of label noise severely compromises the generalization performance of the models. Compared to other types of noise, label noise is considered more harmful to the model’s performance [20]. Learning from datasets with noisy labels has become an important task in modern deep learning applications.

To avoid the model learning incorrect samples, many recent studies have adopted sample selection methods to choose correctly labeled samples from the noisy training dataset. Arpit et al. [21] found that deep learning models tend to first learn from easy samples during the training process and then learn from noisy label samples and difficult samples. Therefore, the small loss selection strategy treats samples with small training losses as clean samples [22]. MentorNet [23], based on the idea of knowledge distillation [24], first uses a teacher model to select clean samples, which are then input into the student model for training, partially avoiding the influence of noisy label samples. Co-teaching [25] proposes using two different models (with different structures or different initializations of the same structure) with different learning abilities to filter out different types of errors caused by noisy labels. Each model selects its own small-loss samples from the same mini-batch and exchanges them with the peer model to update parameters. Co-teaching+ [26] further selects samples with inconsistent predictions from the small-loss samples, encouraging both models to learn the same correct patterns. JoCoR (joint training with co-regularization) [27] uses contrastive loss to measure the consistency of predictions between the two peer models and combines it with the supervised loss of the two peer models to form a joint loss. It selects a certain proportion of small joint loss samples to train the two peer models simultaneously. Yao et al. [28] believe that the proportions of noisy label samples in different mini-batches are different, and using a relatively fixed proportion to select training samples does not reflect the actual situation. They propose using the Jensen–Shannon divergence (ranging from 0 to 1) to measure the difference between predicted results and true labels, which represents the probability of belonging to clean samples. For samples identified as containing noisy labels, they construct two different views to further measure the difference in predictions between the two views and differentiate between in-distribution samples and out-of-distribution samples.

The method of learning from small-loss samples has overall good performance but can accumulate errors due to incorrect selections. In addition, determining the appropriate proportion of small-loss samples remains a challenge. Existing methods mostly directly use the true noise rate of the dataset as the proportion of small-loss samples. However, it is often difficult to obtain the true noise rate of the dataset in reality, making these methods challenging to directly apply to practical problems. To address this issue, we propose a noise rate adaptive learning mechanism without prior conditions, allowing the model to learn the data noise rate during training. We combine this mechanism with JoCoR and design a robust training paradigm called A-JoCoR.

The contributions of this study are summarized as follows: (1) propose a noise rate adaptive learning mechanism without prior conditions. (2) Combine the proposed noise rate adaptive learning mechanism with JoCoR to design the robust training paradigm A-JoCoR. (3) Using AIS data from the Danish Maritime Authority, which includes 80,000 trajectories with eight ship types, each containing 10,000 samples from January to May 2020 within their territorial waters, we demonstrate the effectiveness of the proposed method for ship trajectory classification problems with noisy labels.

The rest of this paper is organized as follows: Section 2 introduces the methods used in this paper. Section 3 demonstrates the effectiveness of the proposed algorithm through its application to AIS trajectories. Section 4 discusses and analyzes the experimental results. Finally, the conclusions are discussed in Section 5.

## 2. Methods

The classification of ship trajectories with noisy labels in this paper consists of three stages: (1) data preprocessing and construction of the trajectory dataset, (2) adding different levels of label noise to the original dataset through a label transformation matrix, and (3) learning the noisy trajectory dataset using the A-JoCoR robust training paradigm.

### 2.1. AIS Data Preprocessing

**Figure 1:**
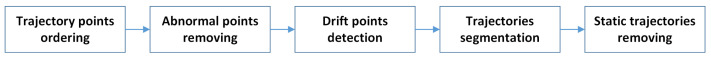
Trajectory preprocessing structure.

AIS data contains a vast amount of information generated by ships during their voyages, comprising a total of 27 fields, including 10 dynamic data fields such as collection timestamp, latitude, longitude, and speed, and 14 static data fields such as ship dimensions, draft, and ship type. Additionally, there are three calculated fields. The names and meanings of some fields are shown in Table 1. Through a survey of existing ship trajectory classification works, this paper ultimately aims to retain seven fields from the AIS data: timestamp, MMSI, latitude, longitude, Speed Over Ground (SOG), Course Over Ground (COG), and ship type. The MMSI serves as a unique identifier for different ship trajectory data, facilitating the segmentation of data from different vessels. The ship type is used as a label for annotating ship trajectory data, and it plays a role in subsequent model training. The combination of timestamp, latitude, and longitude forms a ship trajectory data point containing temporal and spatial information, while the inclusion of SOG and COG enriches the features of the ship trajectory data. However, due to technical malfunctions and coverage limitations, the data may suffer from issues such as missing data and cannot be directly used for training deep learning models. Therefore, preprocessing of the AIS raw data is necessary before constructing the ship trajectory dataset (Figure 1).

The data preprocessing process is as follows. Firstly, the trajectory points in the raw AIS data with the same MMSI are arranged in chronological order. This is performed to separate the navigation trajectories of different vessels. Secondly, trajectory points with missing values or values that clearly do not conform to real-world conditions are removed. This includes cases where the SOG is greater than 80 knots/hour, longitude is greater than 180 ${}^{\circ }$, and latitude is greater than 90 ${}^{\circ }$. Next, a threshold-based method is applied to remove drift points in the trajectories. In this study, a distance threshold of 1 km is set based on experience. If the distance between a trajectory point and the line connecting its preceding and succeeding points exceeds the threshold, the point is considered a drift point and is removed. Furthermore, the trajectories are segmented. A single vessel’s data within a specific time period may include multiple sailing trips. To determine if a vessel’s data within a time period contains multiple sailing trips, time and distance interval thresholds are set for adjacent trajectory points. If the time interval between two adjacent trajectory points exceeds 1 h or the distance interval exceeds 1 km, it is considered that the vessel has started a new trip, and the trajectory is segmented accordingly. After segmenting all trajectory data, the trajectories are further divided into segments of 500 trajectory points each. Trajectory segments with fewer than 500 points and excessively short trajectories are removed, resulting in fixed-length trajectory data of 500 points.

In addition, when analyzing the segmented vessel trajectories, it was observed that many vessels had a prolonged SOG value of 0. Upon observing the latitude and longitude of these trajectory points, it was found that these vessels remained stationary throughout the duration or for a significant period of time. For this category of stationary vessels, it is believed that their trajectories contain insufficient information and cannot be applied to subsequent research tasks. Therefore, their entire trajectories are removed.

After the preprocessing steps described above, this study extracted a total of 381,483 trajectory data points from AIS data within the territorial waters of Denmark from January to May 2020, as provided by the Danish Maritime Authority [29]. These trajectory data points encompassed 15 types of vessel trajectories, as shown in Table 2 with specific information.

It can be observed that the preprocessed ship trajectory data exhibits a severe class imbalance issue. The number of cruise ship trajectories is over two hundred times greater than the number of sailboat trajectories. To ensure a balanced distribution of samples in the constructed dataset, we selects eight types of vessel categories, including passenger ships, tugboats, fishing boats, pilot boats, cargo ships, dredgers, high-speed boats, and search and rescue vessels. Each category consists of 10,000 trajectories, resulting in a total of 80,000 trajectories comprising the ship trajectory dataset for further analysis and research.The training set, validation set, and test set are divided in a ratio of 6:2:2, with the samples of ship trajectories from each class evenly distributed across these datasets.

### 2.2. Noise Label Setting

To evaluate the performance of the proposed method on ship trajectory classification tasks with noisy labels, this study follows the approach outlined in references [27,30,31] to introduce noisy labels into the dataset. Specifically, a label corruption matrix *Q* is employed to intentionally introduce label noise into the constructed dataset.

(1) $$Q_{ij}=\mathrm{Pr}\left[\tilde{y}=j|y=i\right]$$

where $Q_{ij}$ denotes the probability of a clean sample with label *i* being flipped to a noisy sample with label *j*.

**Figure 2:**
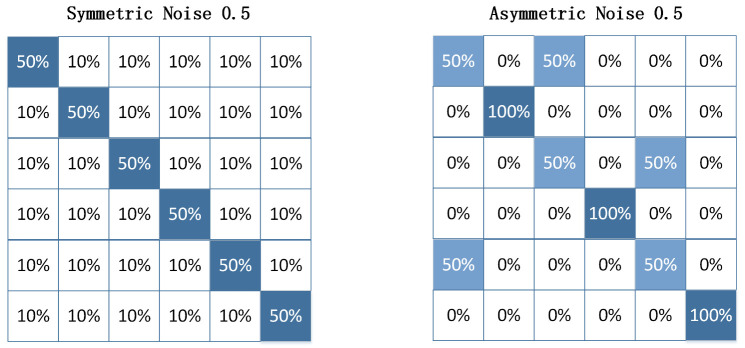
Example of noise transition matrix *Q* (taking six classes and a noise ratio of 0.5 as an example).

In this study, two structures of label corruption matrix *Q* are utilized: (1) symmetric flipping [32], and (2) asymmetric flipping [30], which simulates the noise labels in fine-grained classification. An example of the label corruption matrix *Q* is shown in Figure 2. It is worth noting that asymmetric flipping only selects half of the labels for flipping, resulting in a total label corruption rate that is half of the label flipping ratio.

### 2.3. Robust Training Paradigm for Noise Rate Adaptive Learning

This paper proposes a noise rate adaptive learning mechanism without any prior assumptions. The mechanism enables the model to learn the noise rate of the dataset during the training process, allowing for the adaptive adjustment of the selection ratio of small-loss samples. The proposed mechanism combines this approach with the robust training paradigm JoCoR, resulting in the design of a robust training paradigm called A-JoCoR, which incorporates noise rate adaptive learning.

#### 2.3.1. Mechanism of Noise Rate Adaptive Learning without Prior Assumptions

**Figure 3:**
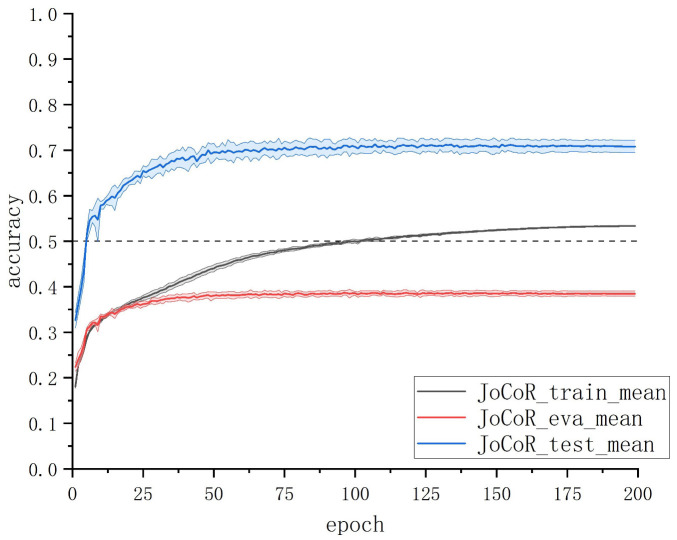
Results of JoCoR on the 50% symmetric noise dataset. Accuracy vs. epochs.

We explored the intrinsic relationship between various metrics of deep learning models during training and the actual data corruption rate. Through experimental analysis on the ship trajectory dataset with noisy labels using JoCoR, taking the results on a 50% symmetric noise dataset as an example (as shown in Figure 3), we found that as the number of training epochs increases, the training accuracy of the model continues to rise until it surpasses the proportion of clean data in the training set. This is because the model gradually fits to the erroneous label data in the later stages of training. However, during this process, the validation accuracy of the model does not rise along with the training accuracy but rather starts to deviate and never exceeds the proportion of clean data. This discrepancy is likely due to inconsistent characteristics of the noise data between the training and validation sets. The features learned from the erroneous label data in the training set do not help improve the model’s classification performance on the validation set, as the relatively easy-to-discriminate clean samples continue to be correctly classified on the validation set.

**Figure 4:**
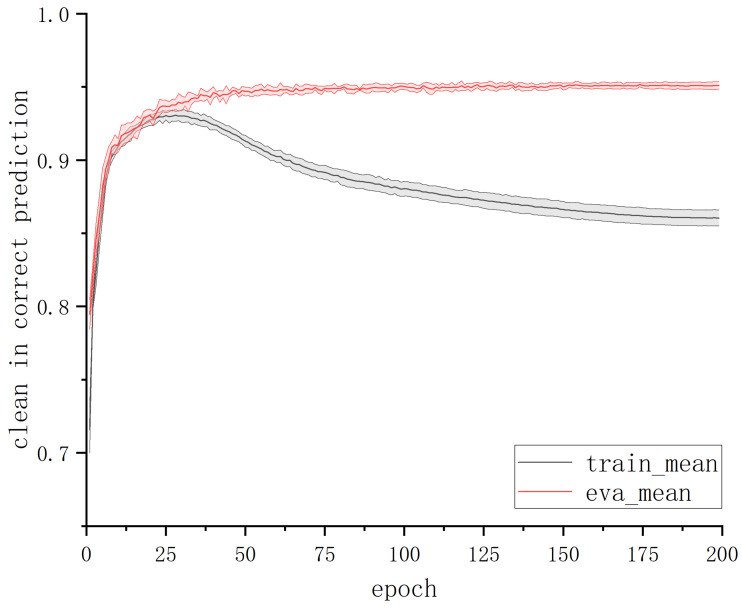
The proportion of clean samples among correctly predicted samples.

To validate this inference, we conducted statistics on the proportion of correctly predicted clean samples in the training set and validation set under each epoch during JoCoR’s training on a 50% symmetric noise dataset. The variation curve of this proportion with respect to epochs is shown in Figure 4. The solid line in the graph represents the mean accuracy over five experiments, and the shaded area represents the STD band.

By comparing Figure 3 and Figure 4, it can be observed that when the training accuracy curve and the validation accuracy curve begin to diverge, the curve representing the proportion of correctly predicted clean samples also starts to separate. The proportion of correctly predicted clean samples in the training set gradually decreases with an increasing number of epochs, while the proportion in the validation set remains relatively unchanged. This alignment perfectly aligns with the changing trends of training accuracy and validation accuracy. This experimental phenomenon confirms the previous inference: the model’s learning of erroneous label data features on the training set does not immediately affect its classification results on the validation set. This also explains why the model’s validation accuracy does not surpass the proportion of clean data.

Based on this finding, we incorporate the model’s validation accuracy into the small-loss sample selection mechanism and propose a no prior condition adaptive learning mechanism for noise rate. The specific design is as follows:

(2) $$\begin{array}{cc} \; & \;R\left(t\right)=1-\tau_{t}\\ \; & \;\tau_{t+1}=\tau_{t}-\rho \left(\tau_{t}-\left(1-\sigma \right)\right)\\ \; & \;\sigma =p_{t}^{eva} \end{array}$$

where *t* represents the training epoch, σ denotes the estimated noise rate of the model in the *t*-th round, $p_{t}^{eva}$ represents the validation accuracy of the model in the *t*-th round, $\tau_{t}$ denotes the sample abandonment rate in the *t*-th round, and the adaptive learning rate $\rho \in \left(0,1\right]$.

The noise rate adaptive learning mechanism proposed in this paper only requires setting the initial values of the sample abandonment rate τ and the adaptive learning rate ρ. With these settings, it can dynamically estimate the noise rate based on the validation accuracy during the model training process. This eliminates the drawbacks of existing robust classification frameworks that rely on prior means to estimate the noise rate.

#### 2.3.2. Robust Training Paradigm for Noise Rate Adaptive Learning

**Figure 5:**
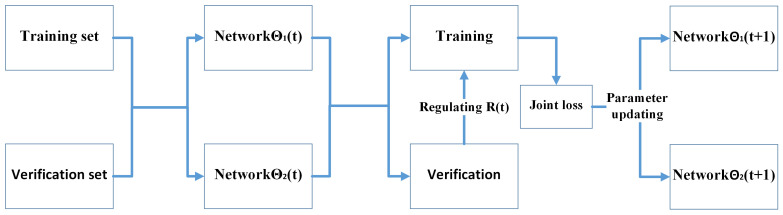
Schematic diagram of A-JoCoR.

To evaluate the effectiveness of the proposed noise rate adaptive learning mechanism, it was combined with JoCoR to introduce a joint training framework called A-JoCoR. The overall framework structure is illustrated in Figure 5.

A-JoCoR initially performs different parameter initializations for two equivalent models with the same structure. Then, during the training process, the noise rate adaptive learning mechanism estimates the true noise rate μ and assigns the estimated noise rate σ to the sample abandonment rate τ. Each equivalent model retains a corresponding proportion of small-loss samples based on the τ value of each epoch. They calculate their respective supervised loss and contrastive loss, forming a joint loss for simultaneous training. This process enhances their discriminative ability towards clean samples and gradually achieves consistent predictions. The detailed procedure is shown in Algorithm 1.
**Algorithm 1** *A-JoCoR***Input:** Network *f* with $\Theta =\{\Theta_{1},\Theta_{2}\}$, learning rate η, training set *D*, epoch $T_{max}$, iteration $I_{max}$, initial sample abandonment rate $\tau_{1}$, the estimated noise rate σ;**for**  $t=1,2,\cdots ,T_{max}$ **do**   Shuffle training set *D*;   **for**  $n=1,2,\cdots ,I_{max}$ **do**     Fetch mini-batch $D_{n}$ from *D*;      $p_{1}=f\left(x,\Theta_{1}\right),\forall x\in D_{n}$;      $p_{2}=f\left(x,\Theta_{2}\right),\forall x\in D_{n}$;     Calculate the joint loss l of $p_{1}$ and $p_{2}$ by Equation (3);     Obtain small-loss sets ${\tilde{D}}_{n}$ by ${\tilde{D}}_{n}={\mathrm{argmin}}_{D_{n}^{\prime }:|D_{n}^{\prime }\left|\geq R\left(t\right)\right|D_{n}|}l\left(D_{n}^{\prime }\right)//$ from $D_{n}$;     Calculate the average loss L of ${\tilde{D}}_{n}$ by $L=\frac{1}{|\tilde{D}|}\sum_{x\in \tilde{D}}l\left(x\right)$;     Update Θ=Θ−η∇L;   **end for**   Obtain $p_{t}^{eva}$;   Update $\sigma =p_{t}^{eva}$;   Update $\tau_{t+1}=\tau_{t}-\rho \left(\tau_{t}-\left(1-\sigma \right)\right)$;   Update $R\left(t+1\right)=1-\tau_{t+1}$;  **end for****Output:**  $w_{f}$ and $w_{g}$

In the context of multi-class classification involving *M* classes, we consider a dataset $D={\left\{x_{i},y_{i}\right\}}_{i=1}^{N}$ consisting of *N* samples, where $x_{i}$ represents the *i*-th instance and $y_{i}\in 1,\cdots ,M$ is its corresponding observed label. Following JoCoR, A-JoCoR involves two deep neural networks referred to as $f(x,\Theta_{1})$ and $f(x,\Theta_{2})$. The prediction probabilities of instance $x_{i}$ are denoted as $p_{1}=\left[p_{1}^{1},p_{1}^{2},\cdots ,p_{1}^{M}\right]$ and $p_{2}=\left[p_{2}^{1},p_{2}^{2},\cdots ,p_{2}^{M}\right]$ for $\Theta_{1}$ and $\Theta_{2}$, respectively. These probabilities are generated by the "softmax" layer outputs of $\Theta_{1}$ and $\Theta_{2}$.

During the training stage of A-JoCoR, each network has the capability to make predictions independently. However, to enhance the collaboration between the networks, a pseudo-siamese paradigm is employed. In this paradigm, although the parameters of the two networks are distinct, they are updated simultaneously using a joint loss. The loss function l, applied to instance $x_{i}$, is constructed in the following manner:

(3) $$l\left(x_{i}\right)=\left(1-\lambda \right)*l_{sup}\left(x_{i},y_{i}\right)+\lambda *l_{con}\left(x_{i}\right)$$

In the loss function, the first part $l_{sup}$ is conventional supervised learning loss of the two networks, the second part $l_{con}$ is the contrastive loss between predictions of the two networks for achieving co-regularization.

For multi-class classification, A-JoCoR use cross-entropy loss as the supervised part to minimize the distance between predictions and labels.

(4) $$l_{sup}\left(x_{i},y_{i}\right)=l_{1}\left(x_{i},y_{i}\right)+l_{2}\left(x_{i},y_{i}\right)=-\sum_{i=1}^{N}\sum_{m=1}^{M}y_{i}\log\left(p_{1}^{m}\left(x_{i}\right)\right)-\sum_{i=1}^{N}\sum_{m=1}^{M}y_{i}\log\left(p_{2}^{m}\left(x_{i}\right)\right)$$

where $l_{1}$ and $l_{2}$ represent the cross-entropy losses of two networks.

Following JoCoR, A-JoCoR incorporates a co-regularization approach by utilizing a contrastive term. This ensures that the two networks guide each other during training. In order to assess the similarity between the predictions of the two networks, A-JoCoR employs the symmetric Kullback–Leibler (KL) divergence.

(5) $$\begin{array}{cc} l_{con} & =D_{KL}(p_{1}\left|\right|p_{2})+D_{KL}(p_{2}\left|\right|p_{1})\\ D_{KL}(p_{1}\left|\right|p_{2}) & =\sum_{i=1}^{N}\sum_{m=1}^{M}p_{1}^{m}\left(x_{i}\right)\log\frac{p_{1}^{m}\left(x_{i}\right)}{p_{2}^{m}\left(x_{i}\right)}\\ D_{KL}(p_{2}\left|\right|p_{1}) & =\sum_{i=1}^{N}\sum_{m=1}^{M}p_{2}^{m}\left(x_{i}\right)\log\frac{p_{2}^{m}\left(x_{i}\right)}{p_{1}^{m}\left(x_{i}\right)} \end{array}$$

where $D_{KL}\left(\right)$ represents the Kullback–Leibler divergence calculation. $D_{KL}(p_{1}\left|\right|p_{2})$ represents the KL divergence from distribution $p_{1}$ to distribution $p_{2}$, while $D_{KL}(p_{2}\left|\right|p_{1})$ represents the KL divergence from distribution $p_{2}$ to distribution $p_{1}$. By summing these two divergences, the overall KL divergence is obtained.

We employ 1D-CNN (1D convolutional neural network) as the network architecture for A-JoCoR, which can be divided into three parts: the input layer, the hidden layers, and the output layer. The input layer of the 1D-CNN transforms the input data into a feature vector x of length 500 with five features. The hidden layers of the 1D-CNN consist of eight 1D convolutional layers with ReLU as the activation function. A 1D max pooling layer follows every two convolutional layers. A basic building block can be formalized as follows:

(6) $$\begin{array}{cc} \; & \;y_{k}=W_{k}⨂x+b_{k}\\ \; & \;h_{k}=ReLU\left(y_{k}\right)\\ \; & \;y_{k+1}=W_{k+1}⨂h_{k}+b_{k+1}\\ \; & \;h_{k+1}=ReLU\left(y_{k+1}\right)\\ \; & \;m_{k}=Maxpooling1d\left(h_{k+1}\right) \end{array}$$

where *k* represents the index of the convolutional layer, k={1,3,5,7}; $W_{k}$ and $b_{k}$ are the weight vector and bias vector for the *k*-th convolutional layer; Maxpooling1d() represents a 1D max pooling operation; ReLU() represents the rectified linear unit activation function applied to the input; ⨂ denotes the convolution operator.

**Figure 6:**
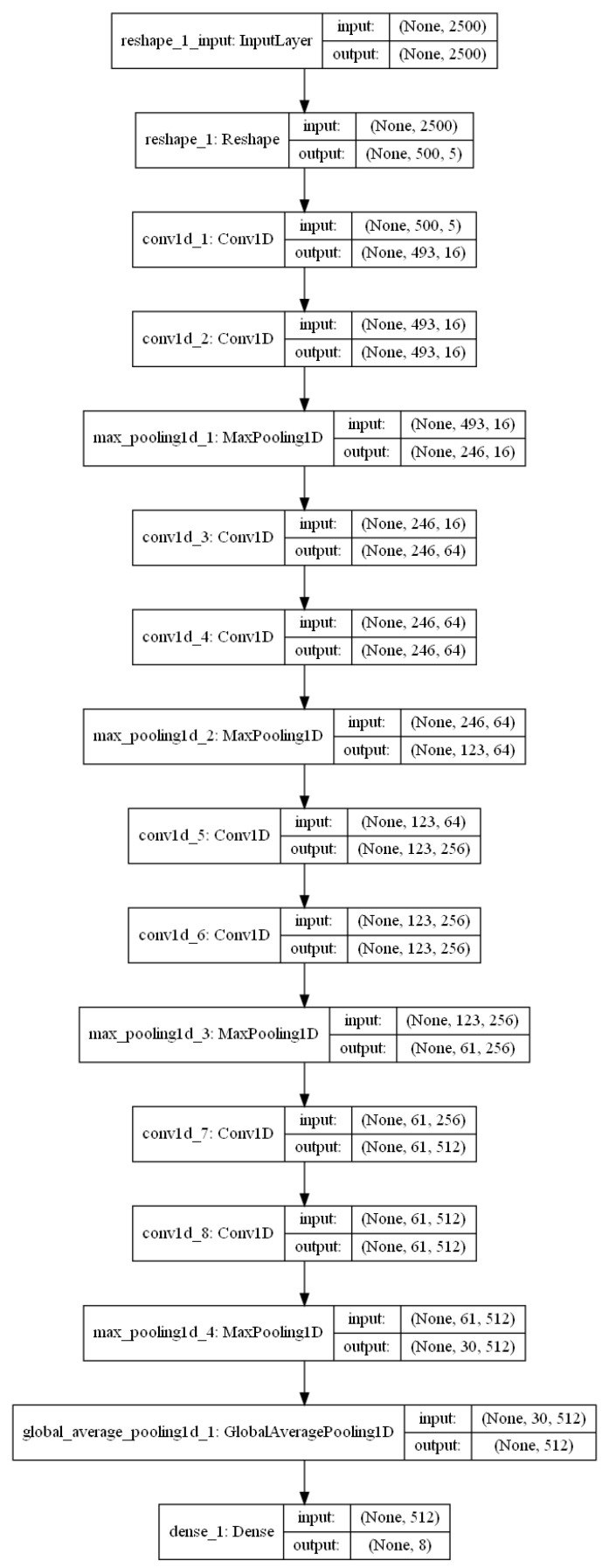
Schematic diagram of the 1D-CNN.

The output layer of the 1D-CNN is composed of a global average pooling layer and a fully connected layer with a softmax activation function, resulting in a probability distribution vector p for the eight ship types. The visualization of the 1D-CNN is shown in Figure 6.

## 3. Results

### 3.1. Experimental Setting

The experiments in this paper were conducted on a single physical machine with the following specifications: Windows 10 operating system, 32 GB of RAM, Intel(R) Core(TM) i7-9850H CPU, and an NVIDIA Quadro T2000 GPU. The experiments were implemented using Python 3.7 programming language and the deep learning framework used was PyTorch.

We used accuracy (acc), precision (*P*), recall (*R*), and F1-score as model evaluation metrics. Accuracy is the proportion of correctly identified samples to the total number of samples. Precision is the proportion of correctly identified samples to the total number of samples identified as that type, which measures the level of correct identification for each type. Recall is the proportion of correctly identified samples to the total number of samples that should have been identified as that type, measuring the completeness of the experimental results. The F1-score is the harmonic mean of precision and recall. Since precision and recall are often conflicting, the F1-score balances the two metrics to simultaneously consider both precision and recall. The calculation formulas for accuracy, precision, recall, and F1-score are as follows:

(7) $$acc=\frac{\sum_{i=1}^{classes=8}correct_{i}}{N}$$

(8) $$P_{i}=\frac{correct_{i}}{Num_{pre_{i}}}$$

(9) $$R_{i}=\frac{correct_{i}}{Num_{true_{i}}}$$

(10) $$F_{1_{i}}=2\times \frac{P_{i}\times R_{i}}{P_{i}+R_{i}}$$

where *i* represents the type of ship, i∈{1,2,⋯,8}; $P_{i}$ represents the precision of samples with the label *i*; $R_{i}$ represents the recall of samples with the label *i*; $F_{1_{i}}$ represents the F1-score of samples with the label *i*; *N* represents the total number of samples; $correct_{i}$ denotes the number of samples where both the label and the model’s classification result are *i*; $Num_{pre_{i}}$ denotes the number of samples where the model’s classification result is *i*; $Num_{true_{i}}$ denotes the number of samples where the label is *i*.

Since ship trajectory classification in our study is a multi-class problem, in order to evaluate the overall performance of the model based on the classification results of all classes, we adopts the macro average method. The macro average is calculated by taking the arithmetic mean of precision, recall, and F1-score for each class. The specific calculation formula is shown below:

(11) $$\begin{array}{cc} \; & \;P_{macro}=\frac{1}{8}\sum_{i=1}^{classes=8}P_{i}\\ \; & \;R_{macro}=\frac{1}{8}\sum_{i=1}^{classes=8}R_{i}\\ \; & \;F_{1_{macro}}=\frac{1}{8}\sum_{i=1}^{classes=8}F_{1_{i}} \end{array}$$

where $P_{macro}$ represents the macro precision; $R_{macro}$ represents the macro recall; $F_{1_{macro}}$ represents the macro F1-score.

After several experimental tests and parameter comparisons, we set the number of epochs for each model to 200, with a learning rate of $5\times 10^{-4}$ and a batch size of 256. Additionally, we configured the hyperparameters $T_{k}$, λ, and τ of JoCoR to replicate the settings described in the original paper, where $T_{k}$ affects the select rate of small-loss samples per epoch in JoCoR, denoted as $R\left(t\right)=1-\min\left\{\frac{t}{T_{K}}\tau ,\tau \right\}$. It is worth noting that in A-JoCoR, we have discarded this hyperparameter and set $R\left(t\right)=1-\tau_{t}$. $T_{k}$ was set to 10, λ was set to 0.1, and τ was set to the actual noise rate in the training set. This configuration signifies that JoCoR initially learns from the entire training set and gradually reduces the proportion of samples it learns from as epochs progress. Starting from the 10th epoch, it exclusively focuses on learning from a proportion of small-loss samples defined by 1−τ, and this proportion remains constant thereafter.

### 3.2. Experimental Design

This section consists of three main parts: (1) experimental testing of the impact of different sample abandonment rate τ values on the classification performance of JoCoR; (2) exploration of the reasonable range for setting the adaptive learning rate ρ in the noise rate adaptive learning mechanism; and (3) solving the ship trajectory classification problem with noisy labels using the A-JoCoR approach based on the 1D-CNN model. The experimental results are compared with JoCoR and 1D-CNN to validate the effectiveness of the proposed method.

#### 3.2.1. The Impact of Noise Rate Estimation on the Effectiveness of JoCoR

To explore the importance of accurately estimating the noise rate for the small-loss sample selection strategy, we take JoCoR as an example. In this experiment, the sample abandonment rate τ is equivalent to the estimated noise rate. We set the sample abandonment rate τ to correspond to 0.1, 0.2, ⋯, 0.8, with an actual noise rate μ of 0.5. Each experiment is repeated five times to test the influence of different estimated noise rates on the JoCoR classification performance under the same noise rate dataset.

**Figure 7:**
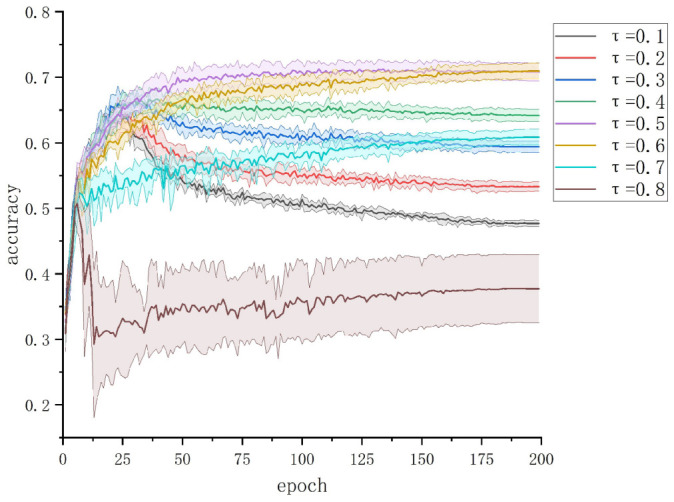
The test accuracy of JoCoR trained on a 50% symmetrically noise dataset for different values of τ.

The test accuracy curves of JoCoR trained on a 50% symmetrically noise dataset for different values of τ are shown in Figure 7. The solid line represents the average accuracy over five experiments, and the shaded area represents the STD band.

It can be observed that estimating the noise rate below or above the actual noise rate of the dataset will both decrease the effectiveness of JoCoR. When the estimated noise rate is lower than the actual noise rate (0.5), the model is prone to overfitting during training due to the presence of a significant proportion of noisy data in the retained samples. As a result, the test accuracy curve shows an initial increase followed by a decrease. The greater the difference between the estimated noise rate and the actual noise rate, the larger the decline in test accuracy. On the other hand, when the estimated noise rate is higher than the actual noise rate, the model has insufficient training data due to a smaller number of retained samples. This leads to poor training performance and significant fluctuations in the results.

It is worth noting that when τ is set to 0.6, it achieves a similar effect to the actual noise rate of the dataset, albeit with a slightly slower convergence speed. This suggests that choosing a slightly higher value of τ than the actual noise rate helps filter out more noisy data without adversely affecting the model’s training performance due to a small number of retained samples. This idea is further supported by subsequent experiments. Therefore, it can be concluded that the selection of the value of τ is crucial for the effectiveness of the robust classification framework, and its appropriate range lies between the actual noise rate of the dataset and a slightly higher value.

#### 3.2.2. The Impact of Adaptive Learning Rate ρ on Model Training

**Figure 8:**
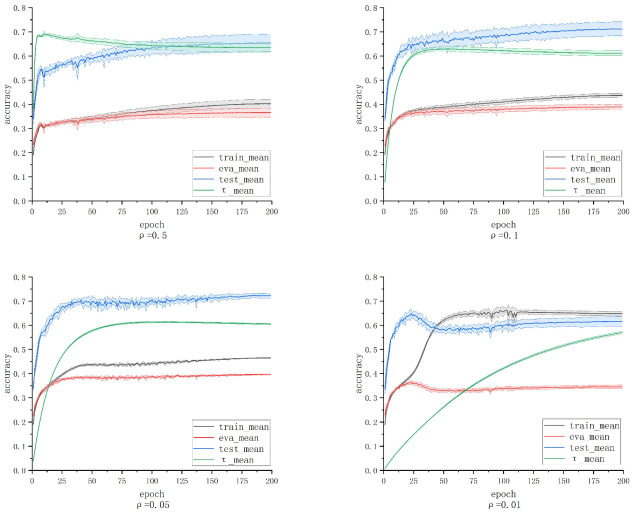
The accuracy and noise estimation curves of A-JoCoR under different ρ settings.

This section explores the reasonable values for the adaptive learning rate ρ through comparative experiments. Following the small-loss sample selection strategy in robust classification frameworks such as JoCoR, we set R(t) to decrease starting from 1 and set $\tau_{1}=0$. We examine the effects of different ρ values, namely 0.5, 0.1, 0.05, and 0.01, on model training. The experiments are conducted using A-JoCoR on a 50% symmetrically noise dataset, repeated five times, with the remaining parameters set the same as in JoCoR. The curves depicting the changes in model training, validation, and test accuracy, as well as the sample abandonment rate τ, for the four different ρ settings, are shown in Figure 8.

It can be observed that when ρ is set to 0.5 and 0.1, the variation curve of the sample abandonment rate τ shows a noticeable trend of initially increasing and then decreasing. This indicates that setting a larger ρ value results in a lower proportion of initially retained samples. As the model’s validation accuracy improves, the model starts to learn from a larger proportion of small-loss samples. On the other hand, when ρ is set to 0.01, the model exhibits significant overfitting in the early stages. This suggests that setting a smaller ρ value delays the growth of τ too much, leading to the inclusion of more noise data in the early retained small-loss samples. When ρ is set to 0.05, the accuracy curve of the model shows an initial increase followed by a decrease and then another increase. We speculate that this may be due to slight overfitting in the early training stages. However, as τ increases, the model’s sample selection proportion decreases, effectively filtering out some noise data that contributes to overfitting. This allows the model to continue learning from clean data. Based on the final results, the model’s accuracy is superior and more stable when ρ is set to 0.05. Therefore, it is concluded that the noise rate adaptive learning mechanism performs well when ρ is set to 0.05.

Based on the above analysis, we conclude that setting ρ to 0.05 allows the noise rate adaptive learning mechanism to gradually filter out noise data for the model without hindering its learning of simple samples in the early stages. Additionally, it enables dynamic adjustment of the proportion of small-loss sample selection based on the model’s training progress.

#### 3.2.3. Comparison of the Performance of A-JoCoR, JoCoR and 1D-CNN

In this section, we conducted five repeated experiments using 1D-CNN, JoCoR, and A-JoCoR on datasets with different levels of noise. These datasets include a 30% symmetric noise dataset, a 50% symmetric noise dataset, a 70% symmetric noise dataset, and a 40% asymmetric noise dataset. We calculated the mean and STD of the experimental results under different conditions.

We retained the output models of 1D-CNN, JoCoR, and A-JoCoR at the best performing epoch in terms of the average validation accuracy on various noise rate datasets. Based on the confusion matrices of these models’ predictions on the test set, macro precision, recall, and F1-score were calculated to evaluate the overall classification performance of the models. The results are shown in Table 3.

**Figure 9:**
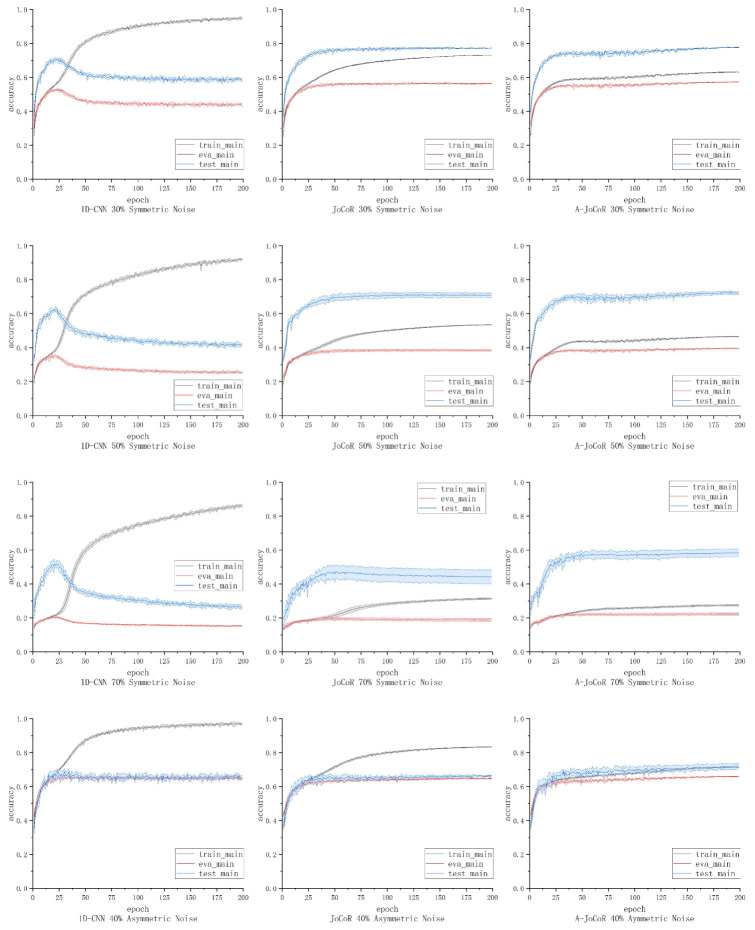
The accuracy curves of 1D-CNN, JoCoR, and A-JoCoR on datasets with different noise ratios.

The training, validation, and testing accuracy curves of the three models on each noise ratio dataset are shown in Figure 9. The solid line represents the mean accuracy of the five experiments, and the shaded area represents the STD. The average test accuracy and STD at the epoch with the best average validation accuracy for each model are shown in Table 4.

From the experimental results, it can be observed that A-JoCoR outperforms the original models on datasets with different noise ratios. In particular, it shows significant improvements on the 70% symmetric noise dataset and the 40% asymmetric noise dataset. Based on this, it can be concluded that the proposed noise ratio adaptive learning mechanism not only allows existing robust learning methods based on small-loss sample selection to adaptively estimate the noise ratio during training, but also significantly enhances model performance, especially in high noise ratio and asymmetric noise scenarios.

## 4. Discussion

To further explain the effectiveness of the noise rate adaptive learning mechanism, we analyzed the noise rate learning effect and the clean sample selection effect of A-JoCoR on various noise ratio datasets.

### 4.1. Noise Rate Learning Effectiveness of the Noise Rate Adaptive Learning Mechanism

**Figure 10:**
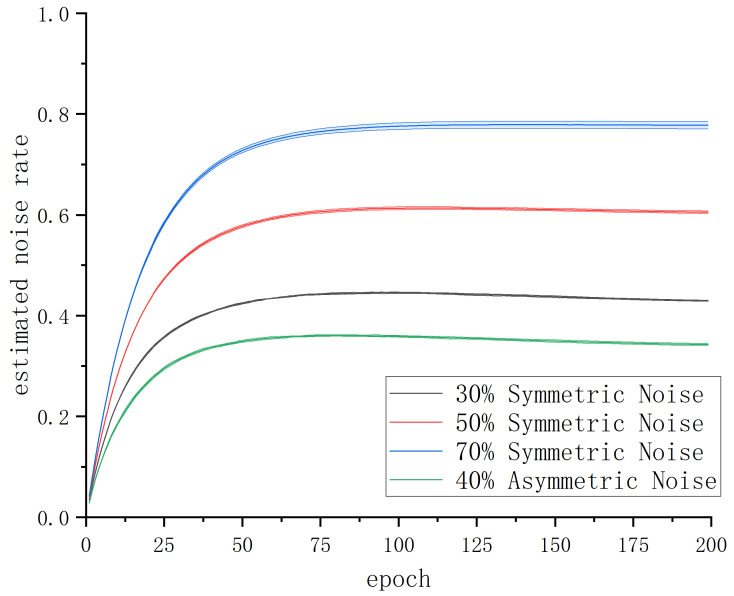
The noise estimation variation curve of A-JoCoR on different noise ratio datasets.

To evaluate the estimation effectiveness of the noise rate adaptive learning mechanism, we conducted statistical analysis on the mean and mean square error of the estimated noise rate σ in five experiments of A-JoCoR on the 30% symmetric noise dataset, 50% symmetric noise dataset, 70% symmetric noise dataset, and 40% asymmetric noise dataset. The results are shown in Figure 10.

It can be observed that the estimated noise rate σ of A-JoCoR has a certain deviation from the true noise rate μ. The estimated noise rate tends to be approximately 10% higher than the true noise rate. However, according to the experimental analysis in Section 3.2.1, when the value of the sample abandonment rate τ is slightly higher than the true noise rate, it does not affect the effectiveness of JoCoR. Therefore, we believe that although there is some error between the estimated noise rate σ and the true noise rate μ in our proposed noise rate adaptive learning mechanism, it is within a reasonable range and does not impact the robustness of the learning process.

### 4.2. Comparison of Clean Sample Selection Effectiveness between A-JoCoR and JoCoR

**Figure 11:**
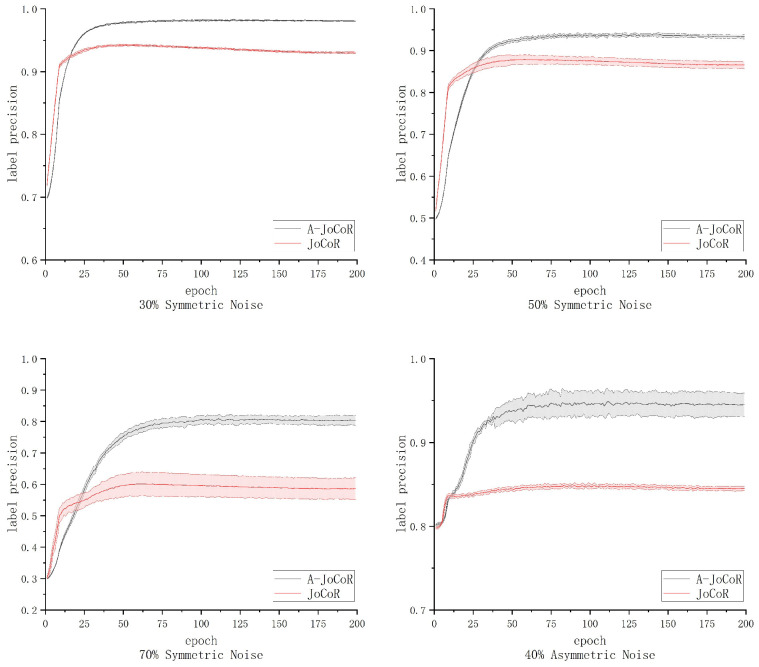
Comparison of clean sample selection effectiveness between A-JoCoR and JoCoR on different noise ratio datasets.

To examine the effectiveness of the noise rate adaptive learning mechanism on the selection of clean samples, we conducted statistical analysis on the average proportion and STD of clean samples selected by A-JoCoR in five experiments on the 30% symmetrically noise dataset, 50% symmetrically noise dataset, 70% symmetrically noise dataset, and 40% asymmetrically noise dataset. A comparison was made with the JoCoR model, which has prior knowledge of the correct noise rates. The comparative results are shown in Figure 11. We use label precision as the evaluation metric for the model’s clean sample selection effect, which is calculated using the following formula:

(12) $$labelprecision=\sum_{i=1}^{I}\frac{clean_{i}}{select_{i}}$$

where *I* represents the number of iterations, $clean_{i}$ represents the number of clean samples selected among the small-loss samples in the *i*-th iteration, and $select_{i}$ represents the total number of small-loss samples selected in the *i*-th iteration.

The comparative results indicate that A-JoCoR improves the filtering effect of noisy data compared to JoCoR in all contamination rate datasets, with particularly significant improvements in the 70% symmetric noise dataset and 40% asymmetric noise dataset, reaching 20% and 10% improvements, respectively. Based on its superior performance, we believe that the contamination rate adaptive learning mechanism can be effectively applied to deep learning problems with noisy labels, providing a significant enhancement to existing robust learning methods based on small-loss sample selection.

### 4.3. Performance Exploration of Noise Rate Adaptive Learning Mechanism in Low-Noise Scenarios

Considering that the number of vessels involved in espionage activities and unlawful behaviors is usually small in real-world scenarios, we further explored the performance of the noise rate adaptive learning mechanism in low-noise scenarios. Specifically, we conducted five repeated experiments on a 10% symmetric noise dataset using 1D-CNN, JoCoR, and A-JoCoR. We retained the output models of 1D-CNN, JoCoR, and A-JoCoR at the best performing epoch in terms of the average validation accuracy. Based on the confusion matrices obtained from these models’ predictions on the test set, we calculated the macro precision, recall, and F1-score, as shown in Table 5.

**Figure 12:**
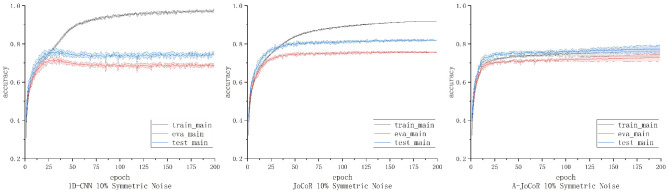
The accuracy curves of 1D-CNN, JoCoR, and A-JoCoR on the 10% symmetric noise dataset.

The training, validation, and testing accuracy curves of the three models on the 10% symmetric noise dataset are shown in Figure 12. The solid line represents the mean accuracy of the five experiments, and the shaded area represents the STD. The average test accuracy and STD at the epoch with the best average validation accuracy for each model are shown in Table 6.

From the above results, it can be observed that in low-noise scenarios, A-JoCoR performs better than 1D-CNN but lower than JoCoR, which already knows the accurate noise rate of the dataset. According to statistical analysis, A-JoCoR estimated the noise rate to be 25.42%, which is higher than the true noise rate of the dataset. This discrepancy between the estimated and true noise rates is the reason behind the lower performance of A-JoCoR compared to JoCoR. When the dataset’s noise rate is high, this deviation may not significantly affect the model’s classification performance. However, when the dataset’s noise rate is at a lower level, this discrepancy could more noticeably affect the model’s performance.

**Figure 13:**
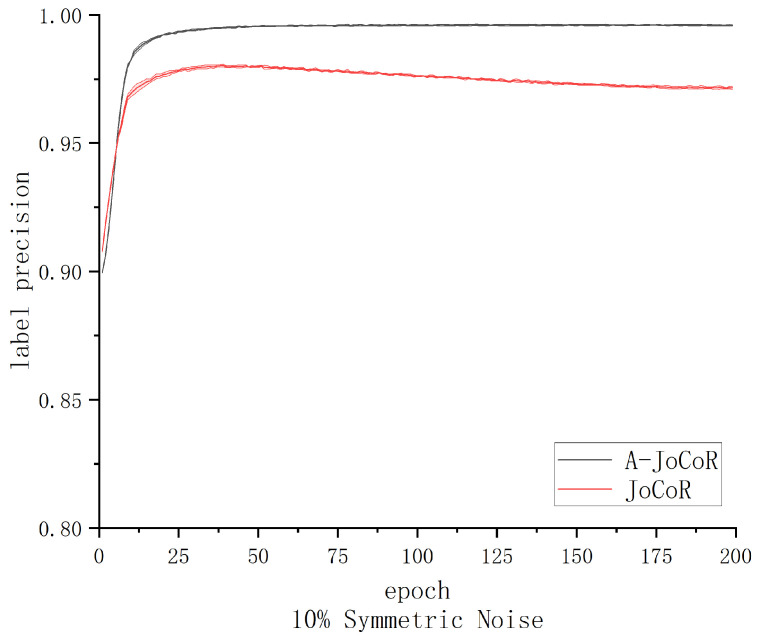
Comparison of clean sample selection effectiveness between A-JoCoR and JoCoR on the 10% noise ratio dataset.

As shown in Figure 13, although A-JoCoR’s performance is lower than JoCoR, which already knows the accurate noise rate of the dataset, in low-noise scenarios, A-JoCoR still exhibits a significant advantage in filtering out noisy data. We speculate that selecting a base model with better classification performance could be beneficial in improving A-JoCoR’s performance in low-noise scenarios. This is because under the premise that the model’s validation accuracy does not exceed the proportion of clean data in the dataset, a higher validation accuracy will help A-JoCoR narrow the gap between the estimated noise rate and the true noise rate, thus enhancing its overall performance.

## 5. Conclusions

AIS data are susceptible to manipulation by vessel owners, and some illicit vessels may intentionally falsify ship type information to conceal illegal fishing activities, espionage operations, or other unlawful behaviors. Additionally, AIS data itself may suffer from data recording or transmission errors, leading to inconsistencies between reported ship type information and the actual situation. This label noise poses challenges for classification tasks based on ship trajectories and poses a serious threat to maritime security.

To address this issue, we proposed a noise rate adaptive learning mechanism without prior assumptions. We combined this mechanism with JoCoR to design a robust training paradigm called A-JoCoR. This paradigm allows the model to adaptively learn the noise rate of the dataset during training, enabling dynamic adjustment of the selection ratio of small-loss samples.

To evaluate the effectiveness of our proposed method on real ship trajectory datasets, we used AIS data published by the Danish Maritime Authority as the original data. Through preprocessing techniques, we constructed a ship trajectory dataset consisting of eight ship types, with 10,000 samples per class and a total of 80,000 trajectories. Extensive experimental results on this dataset demonstrated the effectiveness of our proposed method for ship trajectory classification with noisy labels. Furthermore, thorough ablation studies clearly show that using the noise rate adaptive learning mechanism leads to better clean sample selection effects.

## Figures and Tables

**Table 1:** Partial AIS data fields and their meanings.

Name	Meaning	Example
Timestamp	Timestamp from the AIS basestation	1 January 2020 08:10:43
MMSI	MMSI number of vessel	255806119
Latitude	Latitude of message report	54.70378
Longitude	Longitude of message report	10.888377
ROT	ROT of turn from AIS message if available	−1.1
SOG	Speed Over Ground from AIS message if available	18.6
COG	Course Over Ground from AIS message if available	239.7
Heading	Heading from AIS message if available	240
Ship type	Describes the AIS ship type of this vessel	Pilot
Width	Width of the vessel	4
Length	Length of the vessel	16
Type of position fixing device	Type of positional fixing device from the AIS message	GPS
Draught	Draught field from AIS message	1.7

**Table 2:** The number of preprocessed trajectories for each type of ship.

Ship Type	Number of Trajectories
Passenger	140,715
Fishing	106,149
Pilot	29,329
HSC	25,954
Dredging	19,556
Tug	19,207
SAR	10,982
Cargo	10,822
Tanker	7545
Towing	3196
Law enforcement	2971
Reserved	1799
Anti-pollution	1320
Pleasure	1258
Sailing	680

**Table 3:** The macro precision, recall, and F1-score of 1D-CNN, JoCoR, and A-JoCoR on each noise rate dataset.

Model		$P_{\mathrm{macro}}$	$R_{\mathrm{macro}}$	$F_{1_{\mathrm{macro}}}$
30% Symmetric Noise	1D-CNN	70.29 ± 1.20	69.53 ± 1.21	69.54 ± 1.15
	JoCoR	77.23 ± 0.14	76.94 ± 0.18	77.08 ± 0.15
	A-JoCoR	**78.03 ± 0.29**	**77.61 ± 0.28**	**77.82 ± 0.27**
50% Symmetric Noise	1D-CNN	63.80 ± 3.15	62.44 ± 2.13	62.42 ± 2.84
	JoCoR	71.04 ± 1.36	70.69 ± 1.53	70.86 ± 1.44
	A-JoCoR	**72.58 ± 0.88**	**72.28 ± 0.93**	**72.43 ± 0.89**
70% Symmetric Noise	1D-CNN	51.48 ± 2.09	50.94 ± 2.25	49.76 ± 2.18
	JoCoR	36.32 ± 4.76	46.08 ± 4.03	40.92 ± 4.52
	A-JoCoR	**59.91 ± 2.42**	**58.15 ± 2.22**	**59.01 ± 2.25**
40% Asymmetric Noise	1D-CNN	70.71 ± 1.15	66.79 ± 1.97	65.97 ± 2.30
	JoCoR	70.35 ± 0.69	66.17 ± 0.54	68.19 ± 0.60
	A-JoCoR	**74.12 ± 1.27**	**71.55 ± 1.85**	**72.81 ± 1.55**

**Table 4:** The average testing accuracy and STD of 1D-CNN, JoCoR, and A-JoCoR on datasets with different noise ratios.

Noise Rate	1D-CNN	JoCoR	A-JoCoR
30% Symmetric Noise	69.54 ± 1.20	77.21 ± 0.17	**77.62 ± 0.16**
50% Symmetric Noise	62.48 ± 2.12	70.83 ± 1.36	**72.23 ± 1.01**
70% Symmetric Noise	50.95 ± 2.25	44.24 ± 4.14	**58.24 ± 2.30**
40% Asymmetric Noise	66.74 ± 1.97	66.16 ± 0.45	**70.69 ± 1.73**

**Table 5:** The macro precision, recall, and F1-score of 1D-CNN, JoCoR, and A-JoCoR on the 10% symmetric noise dataset.

Model		$P_{\mathrm{macro}}$	$R_{\mathrm{macro}}$	$F_{1_{\mathrm{macro}}}$
10% Symmetric Noise	1D-CNN	76.85 ± 1.09	75.98 ± 0.80	76.08 ± 0.78
	JoCoR	**81.85 ± 0.37**	**81.66 ± 0.39**	**81.67 ± 0.39**
	A-JoCoR	77.2 ± 1.56	77.24 ± 1.62	77.06 ± 1.58

**Table 6:** The average testing accuracy and STD of 1D-CNN, JoCoR, and A-JoCoR on the 10% symmetric noise dataset.

Noise Rate	1D-CNN	JoCoR	A-JoCoR
10% Symmetric Noise	76.14 ± 0.98	**81.83 ± 0.41**	77.35 ± 1.62

## Data Availability

Not applicable.
